# Editorial: Anaphylaxis – A Distinct Immunological Syndrome, but How Much Do We Really Understand?

**DOI:** 10.3389/fimmu.2019.02943

**Published:** 2019-12-19

**Authors:** Mamidipudi Thirumala Krishna, Margitta Worm, Maria Beatrice Bilo

**Affiliations:** ^1^Department of Allergy and Immunology, University Hospitals Birmingham NHS Foundation Trust, Birmingham, United Kingdom; ^2^Institute of Immunology & Immunotherapy, University of Birmingham, Birmingham, United Kingdom; ^3^Division of Allergy and Immunology, Department of Dermatology, Venereology and Allergy, Charité Universitätsmedizin Berlin, Berlin, Germany; ^4^Allergy Unit, Department of Internal Medicine, University Hospital of Ancona, Ancona, Italy; ^5^Department of Clinical and Molecular Sciences, Polytechnic University of Marche, Ancona, Italy

**Keywords:** anaphylaxis, IgE, tryptase, food allergy, venom allergy, general anesthesia, mastocytosis

The discovery of anaphylaxis as a clinical entity came about in 1903 during Richet and Portier's experiments in laboratory animals for induction of tolerance to actinotoxin (1). They reported some interesting observations, as some animals died soon after receiving a relatively minute dose of the toxin, after having tolerated larger doses previously. The term “anaphylaxis” originates from the Greek word “*aphylaxis”* (opposite to “*phylaxis”* meaning “*protection”*). This was converted to “*anaphylaxis”* as “*phylaxis”* was not a harmonizing or canorous linguistic expression (1).

Despite great strides in allergology and fundamental immunology during the last 2–3 decades, and in an era of personalized and precision medicine, anaphylaxis remains a clinical diagnosis. An important step forward has been the publication of The World Allergy Organization (WAO) clinical criteria for anaphylaxis, which has enabled clinicians across the world to “*speak the same language”* and report meaningful data (2, 3). However, these criteria have been challenged recently campaigning for further refinement (4).

This special edition in *Frontiers in Immunology* (Research Topic—“Anaphylaxis”) embraces some key areas in anaphylaxis, and provides an opportunity to appraise regarding IgE and non-IgE mediated anaphylaxis, immunological mechanisms underlying hymenoptera venom immunotherapy (VIT), clinical utility of serum tryptase measurements in anaphylaxis, novel biomarkers, anaphylaxis in the elderly, refractory anaphylaxis, and peri-operative anaphylaxis during general anesthesia (GA).

The final effector pathway in anaphylaxis is mast cell activation, which culminates into degranulation and release of preformed vasoactive amines, prostaglandins, tryptase, and proinflammatory cytokines that account for mucocutaneous and cardio-respiratory manifestations. Measurement of acute serum total tryptase (AST) is the current gold standard laboratory test for mast cell activation and Beck et al. critically analyze the clinical utility, limitations, and highlight the value of international consensus equation in the diagnosis (5). They also summarize evidence regarding a cautious interpretation of serum tryptase measurements in post-mortem samples and review emerging evidence regarding novel biomarkers such as CCL-2, chymase, carboxypeptidase A3, basogranulin, and platelet activation factor (PAF).

Allergen-specific immunotherapy is effective in IgE mediated allergy including allergic rhinitis, bee, and wasp venom allergy and food allergy (6). It is interesting that despite development of long-term immunological tolerance, vast majority of patients continue to demonstrate sensitization to the respective allergen post-treatment. Sahiner and Durham provide an interesting overview of immunological mechanisms underpinning VIT. The putative mechanisms underlying VIT and other forms of allergen-specific immunotherapy has not been fully elucidated, although research has highlighted the role for allergen-specific Treg/Breg cells, IgG/G4 blocking antibodies, and histamine receptor-2 in mediating peripheral tolerance *via* suppression of Th2 cellular predominance and mast cell activation. Clonal mast cell disorders such as mastocytosis are of great relevance in hymenoptera venom allergy (7). Patients with indolent systemic mastocytosis are usually asymptomatic but develop severe cardiovascular anaphylaxis (with paucity of cutaneous signs and symptoms) following a bee or wasp sting (7, 8). The safety and efficacy of VIT in mast cell disorders has not been well-established (9, 10) but current consensus is to carry out VIT cautiously in those with systemic reactions after demonstrating sensitization to the respective venom (11, 12).

Whilst majority of anaphylaxis is IgE mediated, there are occasional scenarios where there is no evidence of sensitization. Non-IgE mediated anaphylaxis has been proposed as a plausible mechanism involving complement C3a/C5a anaphylatoxins and/or IgG allergen-specific antibodies. Most evidence of non-IgE mediated anaphylaxis comes from studies in animal models. Kow et al. performed a meta-analysis and highlighted role for soluble mediators including histamine, PAF, β-hexosaminidase, IL-6, IL-13, MIP-1α, and TNF-α in IgG anaphylaxis. The main limitation of this report is paucity of publications in this research space.

Food allergy is a leading cause of anaphylaxis in pediatric age group, although not uncommon in adults (2, 3). Several cases of spontaneous anaphylaxis in adults may unfold in time as an IgE mediated allergy to a “hidden allergen.” International migration and travel made human diet more complex due to exposure to diverse allergens and contributed to sensitization to new allergens that may not be native to the patient's geographical area. Multiple episodes of anaphylaxis following consumption of unconnected foods should raise the possibility for a hidden allergen-induced or “summation anaphylaxis” due to co-factor influence. Skypala provides an overview of hidden allergens and influence of co-factors in food-related anaphylaxis. An accurate clinical history with a high index of suspicion is paramount in making a correct diagnosis (Skypala).

Another important development in our understanding of anaphylaxis has been in relation to peri-operative anaphylaxis during GA, refractory anaphylaxis and anaphylaxis in the elderly. Misbah and Krishna provide an overview of peri-operative anaphylaxis and highlight differences in etiology between the UK and France. Recent studies from the UK have shown that latex allergy is exceedingly rare, probably due to implementation of latex free measures in clinical areas. Furthermore, chlorhexidine and patent blue dye have emerged as new allergens in the peri-operative context [Misbah and Krishna; (13)].

Occasionally, anaphylaxis may not respond despite multiple doses of intramuscular adrenaline, i.e., “refractory.” Francuzik et al. analyze data from the European registry in which they report that majority of cases occurred peri-operatively due to drug allergy and identify asthma, multiple co-morbidity, cancer, proton pump inhibitors, aspirin, betablockers, and psychological burden as possible contributing factors.

New therapies enhance longevity, making study of anaphylaxis interesting in the elderly population. Aurich et al. report data on behalf of The Network of Online Anaphylaxis (NORA) in Europe and highlight hymenoptera venom allergy and drug allergy as common precipitants in the elderly, and that anaphylaxis is relatively severe in this age group with cardiovascular involvement.

Whilst anaphylaxis is seemingly a straightforward clinical entity for an acute care physician, its understanding for an allergist is fairly limited at present with respect to factors determining severity, underlying intracellular effector mechanisms within mast cells and basophils, co-factor influence, and immune mechanisms involving of mast cell disorders. Future studies should approach anaphylaxis in a concerted manner with detailed phenotyping, involving multi-center multi-national studies. From a laboratory viewpoint, it is interesting that a small proportion of cases of anaphylaxis show no significant elevation in AST. Further studies are warranted to explore the role for novel biomarkers in serum, urine, and saliva.

## Author Contributions

MK produced the draft manuscript. MK, MB, and MW reviewed, edited, and agreed final version.

### Conflict of Interest

MK received funds from ALK to attend an international conference. His department received educational grants from ALK, MEDA, and Thermo Fisher for PracticAllergy course. MW received compensation for advisory and/or speaker activities from ALK-Abelló Arzneimittel GmbH, Allergopharma, Mylan Germany GmbH, Leo Pharma GmbH, Sanofi-Aventis Deutschland GmbH, Regeneron Pharmaceuticals, Inc., DBV Technologies, Aimmune, Novartis and Eli Lilly. The remaining author declares that the research was conducted in the absence of any commercial or financial relationships that could be construed as a potential conflict of interest.
